# Supplementary material for: The Halotolerant Rhizobacterium—Pseudomonas koreensis MU2 Enhances Inorganic Silicon and Phosphorus Use Efficiency and Augments Salt Stress Tolerance in Soybean (Glycine max L.)
Source: Microorganisms. 2020 Aug 19;8(9):1256. doi: 10.3390/microorganisms8091256 (PMC7570339; doi:10.3390/microorganisms8091256)
Supplement: Supplementary file 1 [file microorganisms-08-01256-s001.pdf]

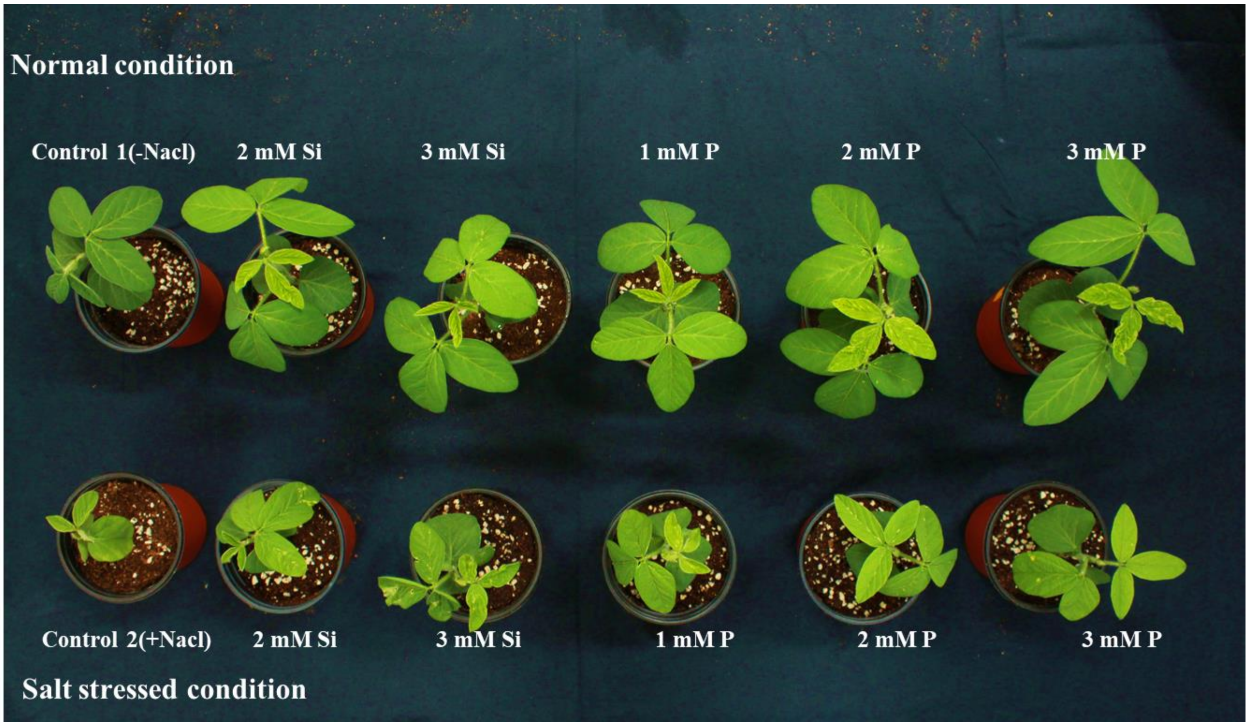

Figure S1. Effect of different doses of silicon and phosphorus on morphological attributes of soybean plants under normal and salt stressed condition.
